# Predicting six-month mortality of patients with traumatic brain injury: usefulness of common intensive care severity scores

**DOI:** 10.1186/cc13814

**Published:** 2014-04-03

**Authors:** Rahul Raj, Markus Benedikt Skrifvars, Stepani Bendel, Tuomas Selander, Riku Kivisaari, Jari Siironen, Matti Reinikainen

**Affiliations:** 1 Department of Neurosurgery, Helsinki University Central Hospital, Topeliuksenkatu 5, PB 266, Helsinki FI-00029, HUS, Finland; 2 Department of Anesthesiology, Intensive Care, Emergency Care and Pain management, Helsinki University Central Hospital, Topeliuksenkatu 5, PB 266, Helsinki FI-00029, HUS, Finland; 3 Department of Intensive Care, Kuopio University Hospital and Kuopio University, Puijonlaaksontie 2, 70211 Kuopio, Finland; 4 Science Service Center, Kuopio University Hospital, Puijonlaaksontie 2, Kuopio, 70211, Finland; 5 Department of Intensive Care, North Karelia Central Hospital, Tikkamäentie 16, 80210 Joensuu, Finland

## Abstract

**Introduction:**

The aim of this study was to evaluate the usefulness of the APACHE II (Acute Physiology and Chronic Health Evaluation II), SAPS II (Simplified Acute Physiology Score II) and SOFA (Sequential Organ Failure Assessment) scores compared to simpler models based on age and Glasgow Coma Scale (GCS) in predicting long-term outcome of patients with moderate-to-severe traumatic brain injury (TBI) treated in the intensive care unit (ICU).

**Methods:**

A national ICU database was screened for eligible TBI patients (age over 15 years, GCS 3–13) admitted in 2003–2012. Logistic regression was used for customization of APACHE II, SAPS II and SOFA score-based models for six-month mortality prediction. These models were compared to an adjusted SOFA-based model (including age) and a reference model (age and GCS). Internal validation was performed by a randomized split-sample technique. Prognostic performance was determined by assessing discrimination, calibration and precision.

**Results:**

In total, 1,625 patients were included. The overall six-month mortality was 33%. The APACHE II and SAPS II-based models showed good discrimination (area under the curve (AUC) 0.79, 95% confidence interval (CI) 0.75 to 0.82; and 0.80, 95% CI 0.77 to 0.83, respectively), calibration (*P* > 0.05) and precision (Brier score 0.166 to 0.167). The SOFA-based model showed poor discrimination (AUC 0.68, 95% CI 0.64 to 0.72) and precision (Brier score 0.201) but good calibration (*P* > 0.05). The AUC of the SOFA-based model was significantly improved after the insertion of age and GCS (∆AUC +0.11, *P* < 0.001). The performance of the reference model was comparable to the APACHE II and SAPS II in terms of discrimination (AUC 0.77; compared to APACHE II, ΔAUC −0.02, *P* = 0.425; compared to SAPS II, ΔAUC −0.03, *P* = 0.218), calibration (*P* > 0.05) and precision (Brier score 0.181).

**Conclusions:**

A simple prognostic model, based only on age and GCS, displayed a fairly good prognostic performance in predicting six-month mortality of ICU-treated patients with TBI. The use of the more complex scoring systems APACHE II, SAPS II and SOFA added little to the prognostic performance.

## Introduction

Comparing mortality rates of patients treated in different ICUs is meaningless if differences in baseline factors affecting outcome, particularly severity of illness or injury, are not taken into account. Therefore, scoring systems that enable quantification of severity of illness are paramount for the evaluation of quality of intensive care [1-4]. Moreover, precise data on severity of illness and the accompanying risk of death are essential in clinical studies [5,6]. However, a scoring system and its associated risk prediction model is useful only if it demonstrates both good calibration and discrimination [7-10].

Among the most commonly used severity of illness scoring systems in intensive care are the APACHE II (acute physiology and chronic health evaluation II) and the SAPS II (simplified acute physiology score II) [11-13]. They were developed for the general ICU population and include equations for the prediction of the risk of in-hospital death [11,12]. Another commonly used scoring system is the SOFA (sequential organ failure assessment) score, which was designed as a measure of organ dysfunction, but has also been used for outcome prediction [14-18].

Patients with traumatic brain injury (TBI) represent a specific type of ICU patients with a substantially different prognosis to the general ICU population [19]. Importantly, in patients with TBI, hospital discharge mortality is a poor outcome measure as it significantly underestimates mortality rates [20]. Thus, using hospital mortality as an endpoint may cause bias if discharge practices differ and models able to adequately predict long-term outcomes are needed [21-23]. The role of the ICU scoring systems for long-term outcome prediction in patients with TBI treated in the ICU is uncertain, and although TBI-specific prognostic models are likely to be more accurate than the ICU scoring systems in this patient group, they are not as widely implemented [13,19]. Therefore, we decided to evaluate the usefulness of the APACHE II, SAPS II and SOFA scores in predicting six-month mortality after TBI and to find out whether these scoring systems are of any additional value compared to a simple model based only on age and the GCS.

## Methods

Using the database of the Finnish Intensive Care Consortium (FICC) we retrieved data on patients who were aged >15 years, and were treated in an ICU with neurosurgical expertise (university hospitals in Finland) during a ten-year period (2003 to 2012). To exclude outcome bias we only included patients with a moderate-to-severe TBI (GCS ≤13) during the first ICU day [24]. The FICC is a high-quality database that prospectively collects data on the characteristics and severity of illness as well as outcomes from ICUs all over Finland [25]. Treatment standards in included institutions are according to the Brain Trauma Foundation guidelines [26]. The Ethics committee of the Northern Savonia hospital district approved the study. As the FICC database is an anonymous database the Ethics committee of Northern Savonia hospital district waived the need for informed consent. Following that, the FICC board granted us access to the database.

### Statistical analysis

We used the χ^2^ test (two-tailed) for categorical univariate analyses. We tested continuous variables for skewness and chose appropriate statistical tests accordingly. We used the Mann-Whitney *U*-test for non-parametric data and the Student *t*-test for parametric data. Parametric data are presented as mean (SD) and non-parametric data as median (IQR). The primary outcome was six-month mortality; a secondary outcome was in-hospital mortality.

To assess the performance of the different scoring systems a split-sample technique was used, where the study population was randomly divided into a development and validation cohort [27]. Logistic regression analysis was used for customization of the APACHE II, SAPS II and SOFA-based prediction models for six-month mortality prediction. The risk of death is calculated using the equation:

$$1/\left(1+e^{‒\mathrm{logit}}\right),$$

where each scoring system has a defined logit (see Additional file 1). For the adjusted SOFA, age was added into the model as an additional variable and the GCS component from the SOFA score was extracted and inserted as a separate variable. A reference model, including only age and the worst measured GCS in the first 24 hours in the ICU, was built for comparison. For the adjusted SOFA and reference models the age and GCS were tested as binominal, categorical and continuous variables. Dichotomization of GCS (based on the median) and ten-year interval age-categorization was found to yield the best results. All models were also customized for in-hospital mortality prediction, in order to assess differences in prognostic performances of each scoring system regarding both short and long-term mortality.

Scoring system performance was assessed by determining discrimination, calibration and precision [28]. Discrimination refers to the ability to separate between those who die and those who survive. It is measured by calculating the area under the receiver operating characteristic curve (AUC). An AUC of 0.50 is no better than mere chance, whereas values > 0.90, >0.80 and >0.70 are considered excellent, good and satisfactory, respectively [10]. The AUC curves were compared to one another using the non-parametric DeLong-DeLong test [29].

Calibration refers to the agreement between predicted and observed mortality across different classes of risk and is usually assessed using the Hosmer-Lemeshow *Ĉ*-test (H-L) [10]. The H-L is similar to the χ^2^ test. The test divides the patients according to the predicted risk of death into equally sized deciles and compares the expected number of deaths to the observed number of deaths in each decile to generate a χ^2^ with an associated *P*-value; the smaller the χ^2^, the bigger the *P*-value and the better the goodness of fit, that is, calibration. A *P*-value >0.05 indicates no significant difference between the predicted and observed outcome and the model is considered well-calibrated [5,10]. However, the H-L test has been criticized for being largely dependent on sample size and thus non-informative in large datasets, and for dividing the patients into deciles, not accounting for the individual patient [7,9]. Furthermore, the classic calibration curves often drawn based on the H-L test are not really curves and should not be used as such (ten dots, which are independent of each other, should not be connected by a line) [30].

To overcome the limitations of the H-L, we combined the classic H-L test with a new statistical test for calibration, the GiViTI calibration belt [30,31]. In addition to giving a calibration curve that illustrates the relationship between predicted risk and observed outcome over different levels of risk, this technique also gives the confidence belt of the curve, that is, an estimation of the degree of uncertainty regarding the true location of the curve. In the GiViTI calibration belt, the relationship between the predicted and observed outcome is calculated by fitting a polynomial logistic function between the logit transformation of the predicted probability and outcome. The calibration belt calculates the 80% CI (light gray area) and 95% CI (dark gray area) surrounding the calibration curve. A statistically significant deviation from the bisector vector (diagonal line for perfect calibration) occurs when the 95% CI does not cover the bisector.

Precision was measured by the Brier score, which is the mean squared difference between the observed and predicted outcome, comprehending both calibration and discrimination [32]. When the incidence of the outcome is 50% the Brier score ranges from 0.0 (perfect) to 0.25 (worthless) [33].

For the statistical analyses, IBM SPSS Statistics 20.0 for Windows and R version 3.0.1 for Windows (R Foundation for Statistical Computing, Vienna, Austria) were used. The H-L calibration was plotted using the PredictABEL library and the calibration belt was plotted using the GiViTI calibration belt library [30,34,35].

## Results

### Baseline characteristics

**Figure 1:**
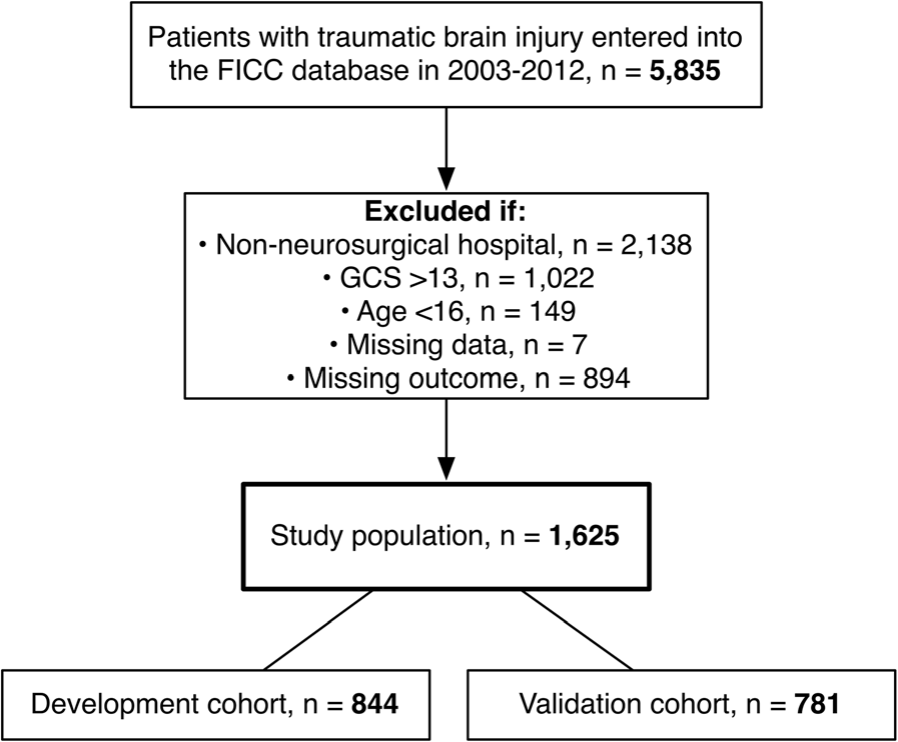
**Study population.** FICC, Finnish Intensive Care Consortium; GCS, Glasgow coma scale.

In total 1,625 patients were included: 844 patients were randomized to the development cohort and 781 to the validation cohort (Figure 1). The median age was 55 years (IQR 38 to 66). Overall crude 6-month mortality was 33% (n = 540/1625); 64% of all deaths (n = 346) took place in the index hospital. There were no significant differences in baseline characteristics, severity scores or outcome between the development and validation cohorts. Differences in scoring system variables between 6-month survivors and non-survivors are shown in Table 1 and in Additional file 2. For the adjusted SOFA and the reference models, the GCS was dichotomized to 3 to 6 and 7 to 13 (based on the median GCS). The relationship and effect of GCS and age on 6-month mortality is shown in Table 2, and illustrated in Additional file 3.

**Table 1:** Baseline characteristics for development and validation cohorts and for six-month survivors and non-survivors

**Variable**	**All patients (n = 1625)**	**Development (n = 844)**	**Validation (n = 781)**	***P*-value**	**Survivors (n = 1085)**	**Non-survivors (n = 540)**	***P*-value**
**Age,** years	55 (38 to 66)	56 (39–66)	54 (36–66)	0.288	52 (33–63)	61 (49–80)	<0.001
**GCS**							
3 to 6	828 (51)	420 (50)	408 (52)	0.318	419 (39)	409 (76)	<0.001
7 to 13	797 (49)	424 (50)	373 (48)		666 (61)	131 (24)	
**APACHE II**	22 (17 to 27)	22 (17 to 22)	22 (17 to 26)	0.784	19 (15 to 23)	27 (22 to 31)	<0.001
**SAPS II**	43 (31 to 55)	43 (32 to 55)	44 (31 to 55)	0.988	38 (28 to 48)	56 (45 to 63)	<0.001
**SOFA**	7 (5 to 10)	7 (5 to 10)	7 (5 to 10)	0.744	7 (5 to 9)	9 (7 to 11)	<0.001
**Length of stay, days**							
ICU	2 (1 to 5)	2 (1 to 5)	2 (1 to 5)	0.989	3 (1 to 6)	2 (1 to 4)	<0.001
Hospital	6 (3 to 12)	6 (3 to 12)	6 (3 to 13)	0.457	7 (4 to 15)	4 (1 to 8)	<0.001
**Mortality**							
ICU	212 (13)	107 (13)	105 (13)	0.647	NA	212 (39)	NA
Hospital	346 (21)	173 (21)	173 (22)	0.416	NA	346 (64)	NA
Six-month	540 (33)	278 (33)	262 (34)	0.795	NA	540 (100)	NA

Categorical variables are presented as number (%), all continuous variables were highly skewed and are presented as median (IQR). APACHE II, acute physiology and chronic health evaluation II; SAPS II, simplified acute physiology score II; SOFA, sequential organ failure assessment; NA, not applicable.

**Table 2:** Relationship between age and Glasgow coma scale (GCS) on six-month mortality

**Age, years**	**Mortality, % (absolute numbers)**		
	**All patients (n = 1625)**	**GCS 7 to 13 (n = 797)**	**GCS 3 to 6 (n = 828)**
<40	20 (86/438)	2 (4/210)	36 (82/228)
40 to 49	27 (50/187)	11 (11/97)	43 (39/90)
50 to 59	32 (116/363)	10 (17/167)	51 (99/196)
60 to 69	42 (129/309)	24 (36/149)	58 (93/160)
70 to 79	45 (104/232)	31 (39/125)	61 (65/107)
≥80	57 (55/96)	49 (24/49)	66 (31/47)

Age groups shown in rows and GCS groups in columns.

### Hospital mortality performance

Following customization and validation the SAPS II showed the highest discriminative power (AUC 0.81), followed by the APACHE II (AUC 0.80), the adjusted SOFA (AUC 0.79), the reference model (AUC 0.74) and the SOFA (AUC 0.73). The AUCs of the SAPS II and APACHE II were significantly higher compared to the reference model (∆AUC 0.07, *P* = 0.011; ∆AUC 0.06, *P* = 0.013, respectively). The adjusted SOFA score showed significantly improved discriminative power compared to the original SOFA score (AUC 0.79 versus 0.73, ∆AUC 0.06, *P* = 0.018).

All models displayed good calibration for hospital mortality prediction with no significant over- or under-prediction intervals, as measured by the GiViTI calibration belt (see Additional files 4 and 5). Precision, as measured by the Brier score, varied between 0.128 and 0.151.

### Six-month mortality performance

**Figure 2:**
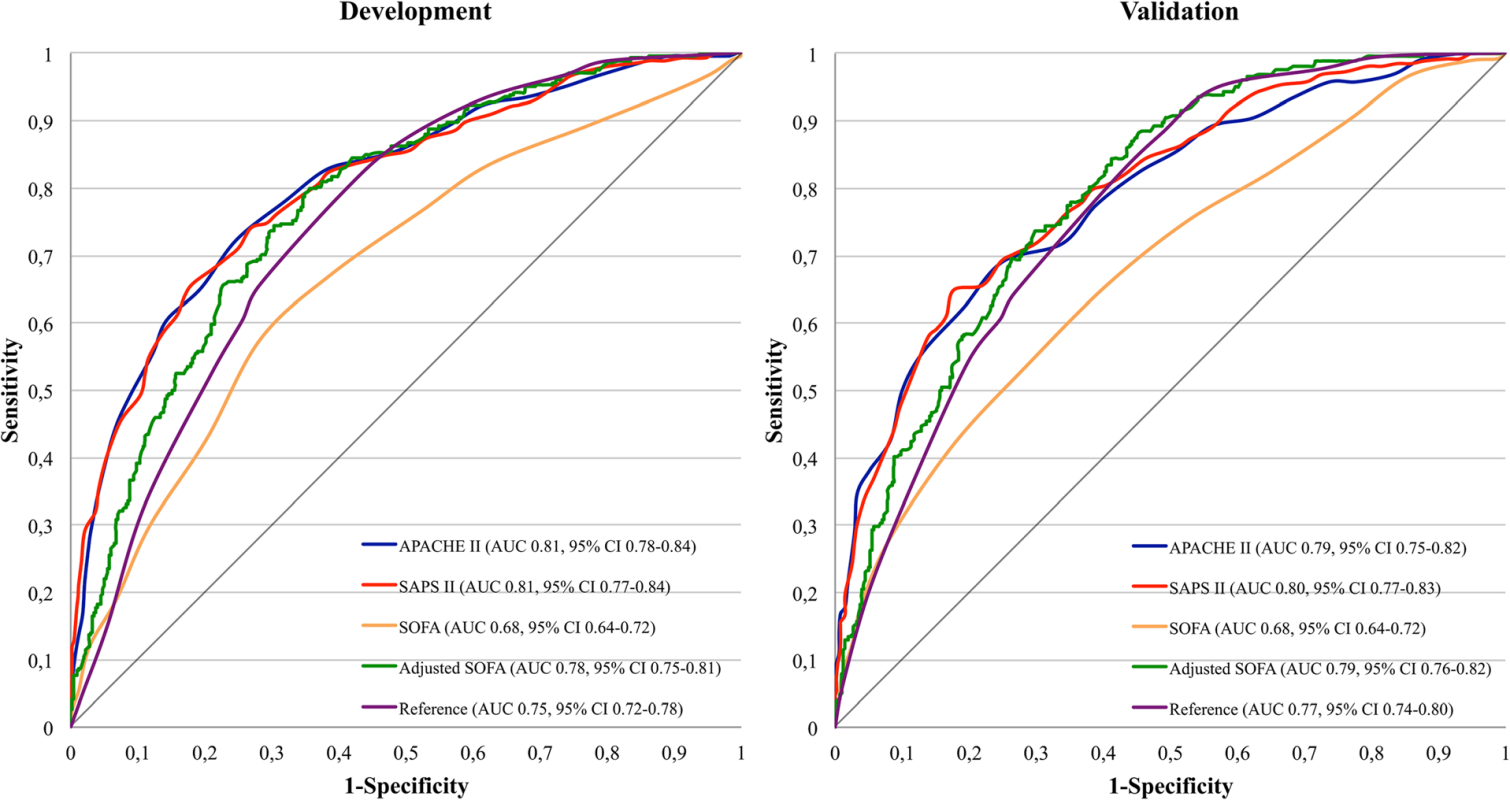
**Area under the curve (AUC) for six-month mortality prediction.** Left panel, the development cohort; right panel, the validation cohort. APACHE II, acute physiology and chronic health evaluation II; GCS, Glasgow coma scale; SAPS II, simplified acute physiology score II; SOFA, sequential organ failure assessment.

The scoring systems showed a lower discriminative power for predicting 6-month mortality compared to predicting in-hospital mortality. Following customization and validation, the discriminative power was highest for SAPS II (AUC 0.80), followed by APACHE II (AUC 0.79), the adjusted SOFA (AUC 0.79), the reference model (AUC 0.77) and the SOFA (AUC 0.68) (Figure 2). The AUC of the SOFA-based model was significantly improved after the insertion of age and GCS (∆AUC 0.11, *P* <0.001) to match the ones of APACHE II (*P* = 0.920) and SAPS II (*P* = 0.745). None of the scoring systems had higher AUCs compared to the reference model (reference model versus APACHE II, *P* = 0.425; reference model versus SAPS II, *P* = 0.218) (Table 3).

**Table 3:** Scoring system performance for six-month mortality

**Performance variable**	**Discrimination**		**Calibration**		**Precision**
	**AUC**	**95% CI**	**H-L *P *-value**	**GiViTI *P *-value**^**‡**^	**Brier score**
**Development cohort**					
APACHE II	0.81	0.78, 0.84	0.153	NA	0.160
SAPS II	0.81	0.77, 0.84	0.343	NA	0.160
SOFA	0.68	0.64, 0.72	0.282	NA	0.201
Adjusted SOFA*	0.78	0.75, 0.81	0.444	NA	0.175
Reference^†^	0.75	0.72, 0.78	0.144	NA	0.185
**Validation cohort**					
APACHE II	0.79	0.75, 0.82	0.062	0.653	0.167
SAPS II	0.80	0.77, 0.83	0.775	0.782	0.166
SOFA	0.68	0.64, 0.72	0.691	0.710	0.201
Adjusted SOFA*	0.79	0.76, 0.82	0.177	0.574	0.174
Reference†	0.77	0.74, 0.80	0.086	0.072	0.181

*Adjusted SOFA with the addition of age and GCS (as a separate variable); ^**†**^reference model including age and GCS; ^‡^the GiViTI is a calibration tool for external analysis and thus only calculated for the validation cohort. Calibration *P*-values >0.05 indicate good calibration. APACHE II, acute physiology and chronic health evaluation II; SAPS II, simplified acute physiology score II; SOFA, sequential organ failure assessment; AUC, area under the curve; H-L, Hosmer-Lemeshow *Ĉ*-test; GiViTI, Italian Group for the Evaluation of Intervention in Intensive Care Medicine; NA, not applicable.

**Figure 3:**
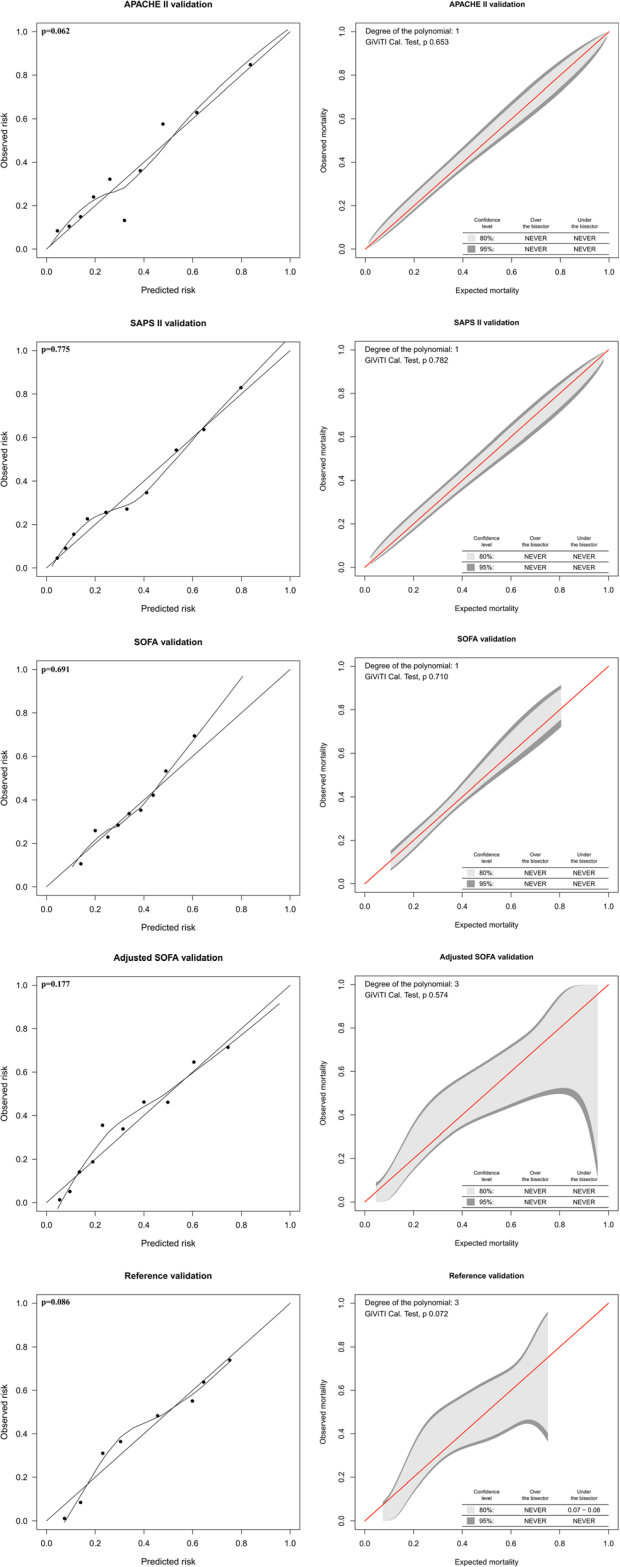
**Calibration for six-month mortality prediction in the validation cohort.** The Italian Group for the Evaluation of Intervention in Intensive Care Medicine (GiViTI) calibration belt (right) and the Hosmer-Lemeshow *Ĉ*-test (H-L) calibration plot (left), with a loess-smoother curve connecting the 10 risk groups. The GiViTI belt visualizes risk intervals of under- and over-prediction, respectively, for a given model as the 95% CI does not cross the red diagonal bisector line.

All models showed good calibration for 6-month mortality prediction according to the H-L test and none of them displayed any significant deviations from the bisector line by the GiViTI tests (*P* >0.05) (Figure 3). Precision, as measured by the Brier score, ranged from 0.166 for SAPS II to 0.201 for SOFA.

## Discussion

### Key findings

We conducted a retrospective study using a nationwide multi-center ICU database, investigating the usefulness of the APACHE II, SAPS II and SOFA scoring systems in predicting long-term mortality for ICU-treated patients with moderate-to-severe TBI. We found that after customization, both the APACHE II and SAPS II-based models showed good performance (discrimination, calibration and precision), whereas the SOFA-based model showed poor performance (poor discrimination and precision but good calibration) for predicting 6-month mortality. The performance of the SOFA-based model was improved with the inclusion of age and the GCS. However, none of these severity score-based models showed superior performance to a simple reference model including only age and the GCS.

### Comparison with previous studies

Previous studies have suggested that APACHE II and SAPS II are either poor or good predictors of short-term mortality in trauma and TBI patients [36-39]. Reports on long-term outcome prediction are scarce. Brinkman *et al*. studied the APACHE IV and found that a customized model had an AUC of 0.84 for predicting 6-month mortality in a non-selected ICU population [23]. In patients with acute myocardial infarction, Huang *et al*. showed the SOFA score to be of moderate value in predicting long-term outcome in patients with acute myocardial infarction (AUC 0.78) [40]. In comparison, the best-performing scoring system in the present study (SAPS II) had an AUC of 0.80.

Similar to the results of Brinkman *et al*., we found the AUCs of the APACHE II- and SAPS II-based models to be higher for predicting in-hospital compared to 6-month mortality [23]. For in-hospital mortality prediction, the SAPS II-based model was significantly superior compared to the reference model. As shown in Additional file 2, initial physiological derangements for patients discharged alive from the hospital were uncommon, whereas they were common for those dying in the hospital. However, among those patients who were discharged alive from the index hospital, there were more evident differences in age and GCS between those who died in the following 6 months and those who survived. Accordingly, the AUC of the reference model was found to be higher for 6-month mortality compared to in-hospital mortality prediction. These findings suggest that abnormal physiology captured by severity of illness scores is of significance for in-hospital but less so for long-term mortality prediction, as long-term outcomes seem to be predominantly determined by age and the GCS.

Compared to the APACHE II and SAPS II, the SOFA score seems to be of limited value in predicting long-term mortality in critically ill TBI patients. One obvious reason for this is that the SOFA score does not pay regard to age, which heavily affects prognosis in this patient group [41]. Furthermore, multiple organ failure (MOF), well demonstrated by a high SOFA score, is a rare complication of TBI, occurring in less than 1% of all TBI patients treated in the ICU [42,43]. In the present study, only one out of 1,625 patients had a SOFA liver score of 4 and 23 patients had a SOFA renal score of 4. Including age and GCS as a separate variable into the adjusted SOFA model improved the discrimination so that the prognostic performance of the model matched that of the best performing models (SAPS II- and APACHE II-based models). This further strengthens the importance of age and GCS in long-term outcome-prediction in this patient group.

To assess calibration we used a new method, namely the GiViTI calibration belt [30]. The GiViTI belt has two main applications: performance comparison between different centers and external validation of prediction models [30,35,44]. Although the mathematical basis of the GiViTI calibration belt has been shown elsewhere it should be compared to the H-L test, which has been considered the gold standard of calibration testing [31,35]. We found the GiViTI and H-L tests to generate similar results for calibration. The main benefit of the GiViTI test is to pinpoint intervals of under- and over-prediction for a given model. Although no significant deviations from the bisector line were noted for any of the models, the reference and the adjusted SOFA models displayed a less-than-perfect calibration belt for 6-month mortality prediction. This is due to the higher degree of polynomial function fitted between the predicted and observed outcome, resulting in wide confidence intervals [35]. This is to our knowledge one of the first clinical studies comparing the traditional H-L calibration test with the GiViTI calibration belt [31]. The GiViTI calibration belt should be strongly considered in further studies in addition to the traditional H-L test.

### Future implications

Future studies should compare the performance of general ICU scoring systems to that of TBI-specific prediction models, such as the IMPACT or the CRASH models [45,46]. In the present study, the best performing scoring system-based models showed AUCs between 0.79 and 0.80 (APACHE II, SAPS II) for predicting 6-month mortality, whereas external validation studies of the IMPACT model have shown AUCs up to 0.87 [47-49]. The general ICU scores lack data on several variables that are important for outcome prediction in TBI patients, for example, pupillary light reaction and head computerized tomography (CT) scan characteristics and signs of increased intracranial pressure, which may reduce predictive ability [45,50,51].

In the era of a more widespread use of TBI-specific prognostic models it is unlikely that future prospective TBI studies will rely only on general ICU scoring systems for case-mix adjustment. However there are numerous high-quality databases in the field of intensive care and trauma medicine around the world that lack some key variables limiting the use of TBI-specific prediction models (for example, the IMPACT). Conversely, general ICU scores (especially APACHE II and SAPS II) are ubiquitously collected in ICUs around the world and based on our results, reliable case-mix adjustment for long-term outcome prediction can be achieved by applying these [13]. On the other hand in epidemiological studies on TBI a simple model including only age and GCS also provides sufficient accuracy. This has implications for case-mix adjustment in forthcoming epidemiological studies [19].

### Limitations

We acknowledge some limitations to our study. First, due to the retrospective nature of the study we were limited to using 6-month mortality as the primary outcome measure. Although mortality is a more clear-cut end point, future studies should consider outcome variables such as neurological outcome and quality of life. Second, as the FICC database does not include radiological data or TBI-specific baseline characteristics, we had to rely on physiological data when evaluating injury severity and could not study the performance of any of the available TBI-specific prediction models, something of key importance for future studies. Third, some long-term outcome data were missing, limiting the power of the study.

## Conclusion

A simple prognostic model, based only on age and GCS, displayed a fairly good prognostic performance in predicting 6-month mortality of ICU-treated patients with TBI. The use of the more complex scoring systems APACHE II, SAPS II and SOFA added little to the prognostic performance.

## Key messages

● The APACHE II and SAPS II-based prediction models showed equally good prognostic performance in predicting 6-month mortality of ICU-treated patients with TBI.

● The SOFA-based model displayed poor performance in 6-month mortality prediction. However, after the inclusion of age and the GCS, as separate variables, the performance improved significantly to match that of the APACHE II and SAPS II.

● A simple prognostic model, including only age and GCS, also displayed fairly good prognostic performance in 6-month mortality prediction.

● Forthcoming epidemiological studies lacking necessary data for the use of TBI-specific models may use the general ICU scoring systems APACHE II and SAPS II or the novel reference model for adequate case-mix adjustment.

## Abbreviations

APACHE II: acute physiology and chronic health evaluation II; AUC: area under the curve; FICC: Finnish Intensive Care Consortium; GCS: Glasgow coma scale; GiViTI: Italian Group for the Evaluation of Intervention in Intensive Care Medicine; H-L: Hosmer-Lemeshow *Ĉ*-test; SAPS II: simplified acute physiology score II; SOFA: sequential organ failure assessment; TBI: traumatic brain injury.

## Competing interests

The authors declare that they have no conflict of interest. The study was funded by a Helsinki University Hospital EVO grant (TYH2012142), *Medicinska Understöds föreningen Liv och Hälsa, Finska Läkaresällskapet* and the Maire Taponen Foundation.

## Authors’ contributions

RR, MS, SB and MR designed the study. MR, SB, MS, JS, RK and RR contributed to the data collection and assembly. RR and TS are responsible for all statistical analyses. All authors contributed to the result interpretation. RR, MR, MS and SB drafted the manuscript. All authors read, edited and approved the manuscript in its final form.

## Supplementary Material

Additional file 1 Scoring system equations for the calculation of 6-month mortality risk. Click here for file

Additional file 2 Table showing scoring system characteristics differences between 6-month survivors and non-survivors. Click here for file

Additional file 3 **Figure showing relationship and effect of age and Glasgow coma scale on outcome.** For the reference and adjusted SOFA models, the GCS was dichotomized to 3 to 6 and 7 to 13, and age was categorized by 10-year intervals (as shown). The figure demonstrates a strong relationship and effect of age and GCS on 6-month mortality. Click here for file

Additional file 4 **Figure showing calibration for in-hospital mortality prediction in the validation cohort.** Right, Italian Group for the Evaluation of Intervention in Intensive Care Medicine (GiViTI) calibration belt; left, traditional Hosmer-Lemeshow *Ĉ*-test (H-L) calibration plot. Click here for file

Additional file 5 Table showing scoring system performance for in-hospital mortality. Click here for file
