# Supplementary material for: Predicting six-month mortality of patients with traumatic brain injury: usefulness of common intensive care severity scores
Source: Crit Care. 2014 Apr 3;18(2):R60. doi: 10.1186/cc13814 (PMC4056363; doi:10.1186/cc13814)
Supplement: Additional file 1 — Scoring system equations for the calculation of 6-month mortality risk. [file cc13814-S1.pdf]

ADDITIONAL FILE 1 - SCORING SYSTEM EQUATIONS

Customized risk for 6-month mortality: $1/(1+e^{-logit})$

- APACHE II logit: -5.183 + APACHE II score * 0.193

- SAPS II logit: -4.967 + SAPS II score * 0.092

- SOFA logit: -2.341 + SOFA day 1 score * 0.209

- Adjusted SOFA logit: -3.008 + SOFA respiration points * 0.031 + SOFA coagulation points * 0.486 + SOFA liver points * -0.038 + SOFA cardiovascular points * 0.044 + SOFA renal points * 0.361 + (1.633, if GCS 3-6) + age points (see below)
  - <40 → 0.0
  - 40-49 → 0.705
  - 50-59 → 0.711
  - 60-69 → 1.153
  - 70-79 → 1.454
  - ≥80 → 1.944

- Reference logit: -2.530 + (1.701, if GCS 3-6) + age points (see below)
  - <40 → 0.0
  - 40-49 → 0.677
  - 50-59 → 0.740
  - 60-69 → 1.231
  - 70-79 → 1.425
  - ≥80 → 1.936

Customized risk for in-hospital mortality: $1/(1+e^{-logit})$

- APACHE II logit: -6.662 + APACHE II score * 0.216

- SAPS II logit: -6.765 + SAPS II score * 0.109

- SOFA logit: -3.459 + SOFA day 1 score * 0.258

- Adjusted SOFA logit: -3.740 + SOFA respiration points * 0.046 + SOFA coagulation points * 0.310 + SOFA liver points * -0.154 + SOFA cardiovascular points * 0.136 + SOFA renal points * 0.392 + (2.267, if GCS 3-6) + age points (see below)
  - <40 → 0.0
  - 40-49 → 0.423
  - 50-59 → 0.304
  - 60-69 → 0.204
  - 70-79 → 0.165
  - ≥80 → 0.404

- Reference logit: -3.135 + (2.380, if GCS 3-6) + age points (see below)
  - <40 → 0.0

- 40-49 → 0.299
- 50-59 → 0.262
- 60-69 → 0.271
- 70-79 → 0.103
- ≥80 → 0.318
