# Supplementary material for: Predicting six-month mortality of patients with traumatic brain injury: usefulness of common intensive care severity scores
Source: Crit Care. 2014 Apr 3;18(2):R60. doi: 10.1186/cc13814 (PMC4056363; doi:10.1186/cc13814)
Supplement: Additional file 2 — Table showing scoring system characteristics differences between 6-month survivors and non-survivors. [file cc13814-S2.docx]

| **Additional file 2:** Scoring system characteristics | | | | |
| --- | --- | --- | --- | --- |
| **Scoring system with variable points** | **Hospital non-survivors** | **Hospital survivors** | | **P-Value*** |
|  |  | **6-month death** | **6-month survival** |  |
| **APACHE II, total score** | 29 (24-33) | 25 (21-28) | 20 (16-24) | <0.001 |
| Age | 2 (0-5) | 5 (3-6) | 2 (0-3) | <0.001 |
| Glasgow Coma Scale | 12 (10-12) | 9 (6-11) | 7 (4-10) | <0.001 |
| Heart rate | 3 (2-3) | 2 (2-3) | 2 (2-3) | 0.252 |
| Mean arterial pressure | 2 (2-3) | 2 (2-2) | 2 (2-2) | 0.008 |
| Temperature | 1 (0-2) | 1 (0-1) | 1 (0-1) | 0.005 |
| Respiratory rate | 1 (0-2) | 1 (0-1) | 1 (0-1) | 0.663 |
| Oxygenation | 0 (0-1) | 0 (0-0) | 0 (0-0) | 0.357 |
| Creatinine | 0 (0-2) | 0 (0-2) | 0 (0-2) | 0.026 |
| Arterial pH | 1 (0-2) | 0 (0-1) | 0 (0-1) | 0.271 |
| Bicarbonate | 2 (0-2) | 0 (0-2) | 0 (0-2) | 0.878 |
| Potassium | 1 (0-1) | 1 (0-1) | 1 (0-1) | 0.122 |
| Sodium | 0 (0-2) | 0 (0-0) | 0 (0-0) | 0.122 |
| White blood cell count | 0 (0-1) | 0 (0-0) | 0 (0-0) | 0.944 |
| Hematocrit | 2 (0-2) | 1 (0-2) | 0 (0-2) | <0.001 |
| Chronic diseases | 0 (0-0) | 0 (0-0) | 0 (0-0) | <0.001 |
| **SAPS II, total score** | 58 (50-64) | 52 (42-60) | 38 (29-48) | <0.001 |
| Admission type | 8 (6-8) | 8 (6-8) | 8 (6-8) | 0.001 |
| Age | 7 (7-12) | 12 (12-16) | 7 (0-12) | <0.001 |
| Glasgow Coma Scale | 26 (26-26) | 13 (7-26) | 13 (5-26) | <0.001 |
| Heart rate | 2 (2-4) | 2 (2-2) | 2 (2-2) | 0.532 |
| Systolic blood pressure | 5 (0-5) | 0 (0-5) | 0 (0-5) | 0.432 |
| Temperature | 0 (0-0 | 0 (0-0) | 0 (0-0) | 0.689 |
| Oxygenation | 6 (6-9) | 6 (0-6) | 6 (0-6) | 0.734 |
| Urine output | 0 (0-0) | 0 (0-0) | 0 (0-0) | 0.506 |
| Urea | 0 (0-0) | 0 (0-0) | 0 (0-0) | <0.001 |
| Bicarbonate | 0 (0-3) | 0 (0-0) | 0 (0-0) | 0.320 |
| White blood cell count | 0 (0-0) | 0 (0-0) | 0 (0-0) | 0.218 |
| Sodium | 1 (0-1) | 0 (0-1) | 0 (0-1) | 0.591 |
| Potassium | 0 (0-3) | 0 (0-0) | 0 (0-0) | 0.115 |
| Bilirubin | 0 (0-0) | 0 (0-0) | 0 (0-0) | <0.001 |
| Chronic diseases | 0 (0-0) | 0 (0-0) | 0 (0-0) | <0.001 |
| **SOFA, total score** | 9 (7-11) | 8 (5-10) | 7 (5-9) | 0.001 |
| Respiration | 2 (1-3) | 2 (1-2) | 1 (1-2) | 0.199 |
| Coagulation | 1 (0-2) | 1 (0-1) | 0 (0-1) | <0.001 |
| Liver | 0 (0-0) | 0 (0-0) | 0 (0-0) | 0.023 |
| Cardiovascular | 3 (1-4) | 1 (0-3) | 1 (0-4) | 0.310 |
| Central nervous system | 4 (4-4) | 3 (2-4) | 3 (2-4) | <0.001 |
| Renal | 0 (0-0) | 0 (0-0) | 0 (0-0) | <0.001 |
| *Comparing the hospital survivors who died within 6-months and those who did not die within 6-months  Values are presented as median (IQR), **Abbreviations**: APACHE II= Acute Physiology and Chronic Health Evaluation II, SAPS= Simplified Acute Physiology Score, SOFA= Sequential Organ Failure Assessment | | | | |
