# Supplementary figures and images for: Predicting six-month mortality of patients with traumatic brain injury: usefulness of common intensive care severity scores
Source: Crit Care. 2014 Apr 3;18(2):R60. doi: 10.1186/cc13814 (PMC4056363; doi:10.1186/cc13814)

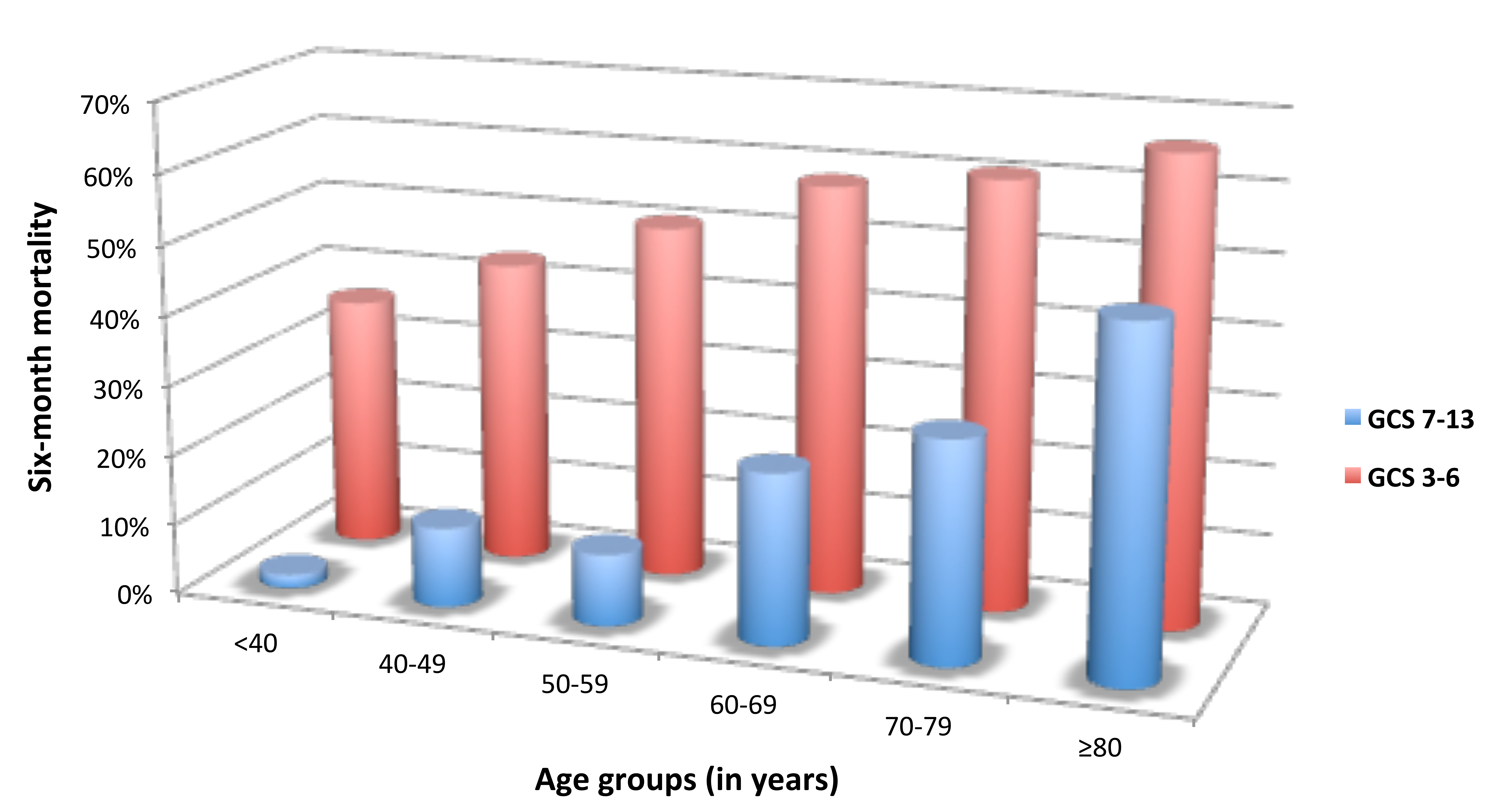

Supplement: Additional file 3 — Figure showing relationship and effect of age and Glasgow coma scale on outcome. For the reference and adjusted SOFA models, the GCS was dichotomized to 3 to 6 and 7 to 13, and age was categorized by 10-year intervals (as shown). The figure demonstrates a strong relationship and effect of age and GCS on 6-month mortality. [file cc13814-S3.tiff]

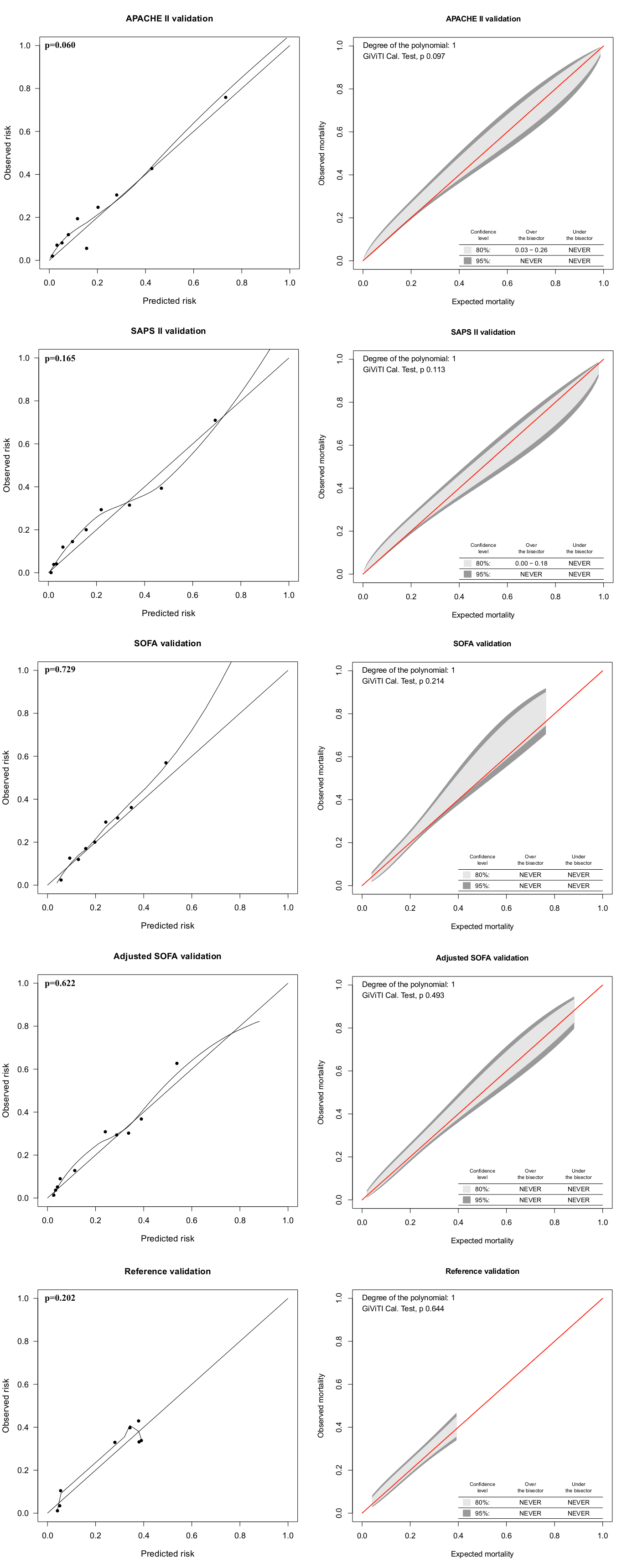

Supplement: Additional file 4 — Figure showing calibration for in-hospital mortality prediction in the validation cohort. Right, Italian Group for the Evaluation of Intervention in Intensive Care Medicine (GiViTI) calibration belt; left, traditional Hosmer-Lemeshow Ĉ-test (H-L) calibration plot. [file cc13814-S4.tiff]
