# Supplementary material for: Predicting six-month mortality of patients with traumatic brain injury: usefulness of common intensive care severity scores
Source: Crit Care. 2014 Apr 3;18(2):R60. doi: 10.1186/cc13814 (PMC4056363; doi:10.1186/cc13814)
Supplement: Additional file 5 — Table showing scoring system performance for in-hospital mortality. [file cc13814-S5.docx]

| **Additional file 5:** Scoring system performance for in-hospital mortality. | | | | | | |
| --- | --- | --- | --- | --- | --- | --- |
| **Performance variable** | **Discrimination** | | **Calibration** | | | **Precision** |
|  | **AUC** | **95% CI** | **H-L p-value** | | **GiViTI p-value**^‡^ | **Brier score** |
| **Development cohort** |  |  |  |  |  |  |
| APACHE II | 0.84 | 0.80 - 0.87 | 0.562 | | - | 0.136 |
| SAPS II | 0.84 | 0.81 - 0.88 | 0.043 | | - | 0.115 |
| SOFA | 0.72 | 0.68 - 0.76 | 0.296 | | - | 0.147 |
| Adjusted SOFA* | 0.83 | 0.79 - 0.86 | 0.174 | | - | 0.122 |
| Reference† | 0.79 | 0.76 - 0.82 | 0.737 | | - | 0.130 |
| **Validation cohort** |  |  |  |  |  |  |
| APACHE II | 0.80 | 0.76 - 0.84 | 0.060 | | 0.097 | 0.128 |
| SAPS II | 0.81 | 0.78 - 0.85 | 0.165 | | 0.113 | 0.131 |
| SOFA | 0.73 | 0.69 - 0.77 | 0.729 | | 0.214 | 0.151 |
| Adjusted SOFA* | 0.79 | 0.76 - 0.83 | 0.622 | | 0.493 | 0.140 |
| Reference† | 0.74 | 0.70 - 0.77 | 0.202 | | 0.644 | 0.149 |
| *****Adjusted SOFA with the addition of age and GCS (as a separate variable), **†**Reference model including age and GCS, ‡The GiViTI is a calibration tool for external analysis and thus only calculated for the validation cohort. **Abbreviations**: AUC= Area Under the Curve, CI= Confidence Interval, H-L= Hosmer-Lemeshow Ĉ-test, GiViTI= Italian Group for the Evaluation of Intervention in Intensive Care Medicine | | | | | | |
